# Urban-rural differences in the health care of people living with dementia and mild cognitive impairment in shared-housing arrangements in Germany – have inequities in urban vs. rural locations been overcome?

**DOI:** 10.1186/s12913-025-12508-z

**Published:** 2025-03-13

**Authors:** Carolin Donath, Antonia Keck, Elmar Graessel, Janissa Altona, Susanne Stiefler, Julia Misonow, Serhat Guenay, Karin Wolf-Ostermann, André Kratzer

**Affiliations:** 1 Center for Health Services Research in Medicine, Department of Psychiatry and Psychotherapy, Uniklinikum Erlangen, Friedrich-Alexander-Universität Erlangen-Nürnberg (FAU), Erlangen, Germany; 2 Institute for Public Health and Nursing Science (IPP), University of Bremen, Bremen, Germany; 3 Competence Center for Clinical Trials Bremen (KKSB), University of Bremen, Bremen, Germany

**Keywords:** Health services research, Dementia, Mild cognitive impairment, Shared-housing arrangements, Urban health services, Rural health services, Health inequities

## Abstract

**Background:**

Previous studies have identified inequities in the diagnostic and therapeutic procedures used with community-dwelling people living with dementia (PlwDs) or people living with mild cognitive impairment (PlwMCIs) depending on the urban vs. rural location of their residence. Whether those differences in health care and health services utilization still exist for people residing in shared-housing arrangements (SHAs) remains unclear at this point.

**Methods:**

In a prospective, multicenter, mixed-methods, cluster-randomized controlled trial, the “DemWG study,” 341 PlwDs or PlwMCIs living in a total of 97 SHAs across Germany were recruited. 31 of the participating SHAs were rural (133 participants), 66 were urban (208 participants). As a secondary analysis we evaluated health care data (e.g. vaccinations, medication), utilization of inpatient/outpatient medical services, non-pharmacological therapies according to the German Remedies Directive, provision of health and medical aids and structural data of the SHAs. Variables were assessed at baseline by trained staff from the SHAs using validated instruments (e.g. FIMA - questionnaire for health-related resource use in an elderly population). Descriptive and inferential statistical methods were applied. *P*-values were corrected with the Benjamini-Hochberg procedure.

**Results:**

The majority of the assessed health care data did not show significant differences between urban and rural SHA inhabitants. After the p-values were corrected, only two variables remained different: inhabitants of rural SHAs were prescribed a significantly larger number of total drugs, while urban inhabitants had significantly more appointments with neurologists/psychiatrists in the last 6 months. There were no significant differences in the use of all other type of inpatient/outpatient services, non-pharmacological therapies, use of health and medical aids. Also, the structural data of the SHAs like staffing did not significantly differ between urban and rural place of living.

**Discussion:**

While it seems that most inequities in the care of PlwDs/PlwMCIs living in SHAs between rural and urban areas have been overcome, there is still the one crucial difference in this non-representative sample of SHAs: the contact with neurologic/psychiatric specialists who offer elaborated diagnostic procedures is less frequent in rural areas.

**Trial registration:**

ISRCTN89825211 (Registered prospectively, 16 July 2019).

**Supplementary Information:**

The online version contains supplementary material available at 10.1186/s12913-025-12508-z.

## Background

Dementia is a complex condition, and the question of how to best care for people living with dementia (PlwDs) or people living with Mild Cognitive Impairment (PlwMCIs) remains one of the largest health and economic challenges nowadays [1–4]. One main focus stressed in the latest World Alzheimer Report is the importance of person-centered care [1]. In addition to being cared for at home by family caregivers, one of the most likely settings for providing this type of care is the shared-housing arrangement (SHA) [5–7]. SHAs are small and homelike care environments that take into account residents’ needs and choices, offer person-centered care, and provide a daily routine that includes meaningful activities of daily living (ADLs). There is some evidence that SHAs can be an advantageous environment for PlwDs and PlwMCIs by improving their quality of life (QoL) and reducing behavioral symptoms of dementia (Donath C, Kratzer A, Graessel E, Keck A, Günay S, Altona J, Misonow J, Stiefler S, Schmidt A, Wolf-Ostermann K: Effect of a complex intervention on agitation and aggression in people living with dementia and mild cognitive impairment in shared-housing arrangements: results of a multicenter, cluster-randomized controlled trial, in preparation) [8].

However, nothing is known about whether the care for PlwDs or PlwMCIs in SHAs is equal in rural vs. urban SHAs. Inequities in diagnostic procedures and quality of care in community-dwelling PlwDs have been reported to depend on living area in Germany [9–11] and internationally [12]. For example, the use of imaging procedures in the diagnostic process was reported to be significantly lower in rural areas in Germany [9]. Similarly, rural inhabitants in the US were less likely to receive neuropsychological testing by psychologists after a diagnosis [13]. A recent systematic review integrating internationally different studies with unique care systems showed that PlwDs in rural areas were– among other differences– hospitalized more often and had a smaller number of visits to general practitioners (GPs) and medical specialists, with the authors interpreting the latter as access barriers [12]. However, even in this large review, only data from PlwDs living at home (with family caregivers or using home care services) or PlwDs living in nursing homes were compared for urbal-rural health-care disparities. In a large claims study of PlwDs in Germany, it was shown that the rate of treatment with anti-dementia drugs depended on the involvement of specialists (neurologists/psychiatrists) such that the probability of a (guideline-adherent) presciption of anti-dementia drugs was twice as high when the person with dementia was either treated only by the named specialist or received a cooperative treatment by a GP and a neuropsychiatric specialist in comparison with the GP-only management of dementia [14]. Given the hypothesis that there might be access barriers to specialists in rural areas [13, 15], it is worth investigating whether there is a difference in guideline adherence concerning anti-dementia treatment between urban and rural areas.

Thus, the aims of this study were:

- to explore whether structural data from the SHAs and staff availability differ between urban and rural SHAs;
- to explore whether there are inequities in diagnoses, medication and vaccination, utilization of inpatient/outpatient medical services, utilization of non-pharmacological therapies according to the German Remedies Directive, and the use of health and medical aids for PlwDs and PlwMCIs living in rural SHAs versus those living in urban SHAs across Germany;
- to investigate whether adherence to the recommendations of the recently (at the end of 2023) published German S3 guidelines on dementia [16] concerning medical treatment of cognitive symptoms differs significantly between rural and urban SHAs.

## Methods

### Study design

The DemWG study was a prospective, multicenter, mixed-methods, cluster-randomized controlled trial with a waitlist control group design [17]. The trial was conducted in 97 SHAs located in 10 different German federal states between April 2019 and December 2022. The data analyzed in this paper were collected at baseline (t0) and constitute a secondary analysis of the health services utilization of people living in SHAs across Germany. All procedures were approved by the Ethics Committee from the University of Bremen (Ref. 2019-18-06-3). In each participating SHA, on-site study coordinators (usually nursing staff) underwent a 4-hour training on the study protocol, data collection, and screening process to conduct the screening, collect the data, and act as a contact person between the study headquarters and the SHA. Participation in the study was voluntary, and both participants and clusters were free to leave the study at any time without repercussions. Written informed consent was obtained from each participant or, if applicable, their legal guardian. The study was registered prospectively on July 16, 2019 in the ISRCTN registry (Trial identification number: ISRCTN89825211). Additional information about the study design can be found in the study protocol [17].

### Recruitment and sample size

The SHAs were recruited between July 2019 and December 2020 in all the federal states in Germany. The participating SHAs were located in Bavaria, Baden-Württemberg, Berlin, Brandenburg, Bremen, Mecklenburg-Vorpommern, Niedersachsen, Rheinland-Pfalz, and Schleswig-Holstein. SHAs and their outpatient care services were identified by their websites or entries in information systems and databases. Furthermore, written information about the study was sent to ministries and administrative authorities from the different German federal states, nursing care centers (Pflegestützpunkte), and German Alzheimer Societies with a request to forward it to the SHAs. All interested SHAs were interviewed by telephone in order to include SHAs with at least three PlwDs or PlwMCIs and to exclude SHAs with a focus on other mental disorders or intensive care before randomization (used two questions see Supplementary file 1). All SHAs interested in participating and meeting the inclusion criteria signed a cooperation agreement. At baseline, 97 SHAs with 341 participants were included at baseline. In line with the German Federal Institute for Research on Building, Urban Affairs, and Spatial Development [18], communities with populations of less than 5,000 and without basic central functions (e.g. libraries, schools, physicians, police, etc.) were classified as rural, and communities with populations of 5,000 or more and communities with basic central functions as urban. 31 of the participating SHAs were rural (133 participants), and 66 were urban (208 participants).

### Eligibility of participants

All residents of each participating SHA were screened. Inclusion criteria were a psychometric verification of mild-to-moderate dementia (i.e. Mini-Mental State Examination [MMSE] < 24) or MCI (i.e. MMSE > 23, but Montreal Cognitive Assessment [MoCA] < 24). Exclusion criteria were: severe dementia (i.e. MMSE < 10) at the time of screening, severe hearing impairment, severe visual impairment, cognitive decline due to diseases other than dementia (e.g. schizophrenia or Korsakoff syndrome), permanent immobility, no verbal communication in German possible, history of more than one stroke, or history of severe major depression. The median (Mdn) time interval between the screening and the baseline data collection was 3 months (Range: 0 to 13 months) because the study had been interrupted by the outbreak of the COVID-19 pandemic in spring 2020. Therefore, although the degree of cognitive impairment at t0 corresponded to severe dementia for 30 individuals, these individuals had been classified as having moderate (*n* = 26) or mild dementia (*n* = 4) at screening and were therefore included in the study in accordance with the study protocol.

### Instruments

Trained nursing staff from the participating SHAs collected the data by means of pseudonymized paper case report forms (CRFs). These CRFs were sent to the data monitoring committee at the Competence Center of Clinical Trials at the University of Bremen (KKSB), where the data were checked for plausibility and completeness. The following instruments that were relevant to the present work were administered.

From the nursing documentation, data concerning medically confirmed dementia diagnoses, prescribed medication, infections, vaccination status, falls, hospitalizations, and sociodemographic data (e.g. age, sex) were drawn. The “FIMA - Questionnaire for health-related resource use in an elderly population, German version” [19] was used to assess the utilization of health services in the last 6 months (inpatient/outpatient medical services, non-pharmacological therapies according to the German Remedies Directive [i.e. medical chiropody, physiotherapy, occupational therapy, speech/language/swallow therapy, other non-medical therapies, e.g. nutrition therapy], prescribed health and medical aids). Its reliability and validity have been confirmed [20]. Besides data on the individual level, structural data concerning the SHAs were also collected. These data included the number of residents in each SHA, the number of GPs serving the SHA, and the number and qualifications of available SHA staff. Furthermore, the structural quality characteristics that were implemented, such as the availability of dementia-specific training concepts, were assessed.

To describe the participants, the following variables are presented in the Results section:

- Cognitive status: Mini-Mental-Status Examination (MMSE) [21].
- Activities of daily living (ADLs): Barthel-Index (BI) [22, 23].
- Comorbidities: Charlson Comorbidity Index (CCI) [24].
- Agitation and Aggression: Cohen-Mansfield Agitation Inventory-Short Form (CMAI-SF) [25].
- Behavioral and Psychological Symptoms of Dementia (BPSD): Neuropsychiatric Inventory (NPI-NH) [26, 27].
- Quality of Life (QoL): Care-specific Quality of Life of people with dementia (QUALIDEM) [28, 29].

The MMSE [21], used to assess the status of participants’ cognitive functions, is the most widely used cognitive screening test for dementia, and its reliability and validity have been established [30–32]. Its scores range from 0 to 30, with higher scores indicating higher cognitive functioning. The Barthel Index [22, 23], used to assess ADLs via proxy ratings, consists of 10 items with a total score ranging from 0 to 100. It is a widely used, reliable, and valid instrument [22, 23, 33], with higher scores indicating better ADL capabilities. The CCI [24] gives a numerical reflection of patients’ overall comorbidity by weighting 12 medical diagnoses by their mortality-associated severity, thus resulting in a total score ranging from 0 to 24. Higher scores depict an increased 1-year mortality. Its validity and reliability have been established [24, 34–36]. The German version of the CMAI-SF [25], assessing agitation and aggression, is a 14-item proxy-based rating concerning the evidence and frequency of verbal and physical agitation and aggressive behaviors derived from the 29-item original CMAI [37, 38]. Scores range from 14 to 70 with higher scores indicating more pronounced agitation and aggression. The reliability and validity of the CMAI-SF [25, 39, 40] have been confirmed. The German version of the NPI-NH [26, 27] is a proxy-based rating of the frequency and severity of 12 BPSDs. It is one of the most widely used instruments in this area [41, 42]. Scores can vary from 0 to 144, with higher scores indicating a higher symptom load. Validity and reliability have been ascertained [26, 41, 43, 44]. The QUALIDEM [28, 29], a proxy-based dementia-specific QoL rating, covers 37 items loading on nine dimensions of QoL. It results in a global score ranging from 0 to 100 [45], with higher scores indicating a higher quality of life. Numerous studies have confirmed its reliability and validity [28, 29, 46–49].

### Statistical analysis

Data analyses were performed with IBM SPSS Statistics, version 28. Single missing values were imputed via iterative random forest imputation. For health service utilization data obtained with the FIMA [19], data that were not explicitly identified as having taken place were counted as a non-utilization/non-offer. We used descriptive and inferential statistical methods to conduct the rural-urban comparisons. The underlying assumption of normal distribution for parametric tests was checked with the Shapiro-Wilk-Test test for each metric variable. It showed that all metric variables were not normally distributed (*p* <.05). Therefore, non-parametric Mann-Whitney-U-tests were used instead of parametric t-tests. The Chi-square test was used for categorial variables. An alpha rate of less than 5% was considered indicative of statistical significance. However, because we computed multiple tests of significance, we adjusted for multiple testing to account for potential inflation of the alpha-error by applying the Benjamini-Hochberg procedure [50] to the data set, as this method controls the false discovery rate more efficiently than the simple Bonferroni method [51]. To assess guideline adherence regarding anti-dementia medication, we systematically checked nine constellations that were either suggested () or not suggested () by the most recent German S3 guidelines [16]. We analyzed the frequency of adherence/non-adherence for each of the nine test cases in an urban-rural comparison. In detail, they were:

1. Acetylcholinesterase inhibitor intake for participants with mild dementia 
2. Acetylcholinesterase inhibitor intake for participants with moderate dementia 
3. Acetylcholinesterase inhibitor intake for participants with severe dementia 
4. Acetylcholinesterase inhibitor intake for participants with MCI 
5. Memantine intake for participants with moderate dementia 
6. Memantine intake for participants with severe dementia 
7. Memantine intake for participants with mild dementia 
8. Memantine intake with parallel intake of Acetylcholinesterase inhibitor 
9. Memantine intake for participants with MCI 

For the analysis regarding anti-dementia medication in urban vs. rural SHAs, the classification of the participants into dementia severity categories was applied as suggested in the German S3 guidelines [16] (MMSE-score 0–9: severe dementia, MMSE-score 10–19: moderate dementia, MMSE-score 20–26: mild dementia).

## Results

### Characteristics of the SHAs

Of the 97 SHAs participating in the study, the average number of available places for care-dependent people per SHA was 10.1 (SD = 2.3; Mdn = 10.0). The number of places most often reported was 12 (32.5%). Furthermore, the average number of inhabitants was 9.5 (SD = 2.3; Mdn = 9.0). The largest number of inhabitants reported was eight people (26.5%). Most of the SHAs (96.9%) were served by one nursing care service (Mdn = 1.0), but the range in the number of involved care services per SHA was one to four (M = 1.1; SD = 2.8). The people living in the SHAs were mostly (32.0%) cared for by two different GPs per SHA (M = 2.0); however, the number of GPs involved ranged from one to eight (M = 2.8; SD = 1.6).

### Comparison of urban and rural SHAs

All of the structural characteristics of the SHAs were not significantly different between urban (*n* = 66) and rural (*n* = 31) SHAs after correction of the p-values (see Table 1).

**Table 1:** Urban-rural comparisons of the SHAs’ structural characteristics

Variable	Mean (SD) / *N* (Percentage) Urban (*n* = 66)	Mean (SD) / *N* (Percentage) Rural (*n* = 31)	*p*-value^a^	corrected *p*-value^b^
Number of inhabitants per SHA	9.22 (2.38)	10.07 (1.96)	0.045*	0.283
Number of available places per SHA	9.91 (2.31)	10.41 (2.23)	0.167	0.497
Participating PlwDs/PlwMCIs in the study	2.92 (1.60)	3.65 (2.18)	0.173	0.497
Number of care services per SHA	1.05 (0.37)	1.06 (0.25)	0.202	0.516
Number of general practitioners per SHA	2.48 (1.32)	3.58 (1.82)	0.003**	0.069
Staff h/day “geriatric nursing specialist with an additional gerontopsychiatric qualification”	1.43 (2.41)	0.50 (1.69)	0.098	0.423
Staff h/day “geriatric nursing specialist without an additional gerontopsychiatric qualification”	8.96 (8.53)	11.15 (27.57)	0.281	0.639
Staff h/day “nursing professional with an additional gerontopsychiatric qualification”	0.49 (1.61)	4.88 (19.36)	0.439	0.797
Staff h/day “nursing professional without an additional gerontopsychiatric qualification”	3.51 (3.00)	7.72 (15.76)	0.158	0.497
Staff h/day “geriatric nursing assistant with an additional gerontopsychiatric qualification”	3.88 (8.64)	3.89 (7.99)	0.981	0.991
Staff h/day “geriatric nursing assistant without an additional gerontopsychiatric qualification”	18.09 (17.61)	21.29 (26.97)	0.991	0.991
Staff h/day “social service voluntary”	0.83 (3.07)	0.68 (2.11)	0.843	0.986
Staff h/day “volunteers”	0.52 (1.07)	0.75 (1.75)	0.739	0.986
Staff h/day “other staff”	9.17 (12.67)	8.20 (10.76)	0.796	0.986
Type of SHA (integrative)^c^	26 (39.4)	19 (61.3)	0.125	0.479
SHAs with a certified nursing care focus on “Dementia”	27 (40.9)	12 (38.7)	0.837	0.986
SHAs with dementia-specific training	44 (66.7)	15 (48.4)	0.085	0.414
SHAs with specialized care for long-term outpatient care	48 (72.7)	22 (71.0)	0.857	0.986

^a^*p*-values for Chi-square tests for categorical variables and Mann-Whitney-U-Test for metric/interval-scaled variables

^b^*p*-values corrected for multiple testing by the Benjamini-Hochberg procedure

^c^ integrative = PlwDs living with other care-dependent residents in one SHA vs. segregated = SHAs designed only for people living with dementia or MCI

Significance level: *p<0.05; **p<0.01

### Characteristics of the participants living in urban vs. rural SHAs

The participants were on average 82.75 years old (SD = 8.44), and the majority (76.2%) of them were female. After the p-values were corrected, there were no significant differences in patient characteristics between those living in urban and rural locations (see Table 2).

**Table 2:** Baseline sample characteristics of the participants

Variable	Mean (SD) / *N* (percentage) Urban (*n* = 208)	Mean (SD) / *N* (percentage) Rural (*n* = 133)	*p*-value^a^	Corrected *p*-value^b^
Age (in years)	82.99 (8.46)	83.27 (8.45)	0.757	0.986
Gender (female)	162 (77.9)	98 (73.7)	0.374	0.759
ADLs (Barthel-Index)	67.21 (25.88)	63.87 (25.77)	0.198	0.516
Agitation and aggression (CMAI-SF)	19.34 (6.45)	18.33 (7.25)	0.006**	0.079
Cognitive status (MMSE)	18.05 (6.11)	19.62 (6.70)	0.008**	0.079
Dementia severity^c^			0.017*	0.117
MCI	19 (9.1)	22 (16.5)		
Mild dementia	59 (28.4)	48 (36.1)		
Moderate dementia	113 (54.3)	50 (37.6)		
Severe dementia	17 (8.2)	13 (9.8)		
Comorbidities (Charlson-Index)	3.16 (1.73)	3.70 (2.70)	0.090	0.414
BPSD (NPI-NH)	10.07 (12.99)	8.28 (12.30)	0.194	0.516
Quality of Life (QUALIDEM)	75.37 (13.05)	79.08 (14.00)	0.005**	0.079
Medical dementia diagnosis (yes)	165 (79.3)	89 (66.9)	0.010*	0.086

^a^*p*-values for Chi-square tests for categorical variables and Mann-Whitney-U-Test for metric/interval-scaled variables

^b^*p*-values corrected for multiple testing by the Benjamini-Hochberg procedure

^c^ according to the new German S3 guidelines on dementia [ 16 ], MCI = MMSE > 26 & MoCA < 24, mild dementia = MMSE 20–26, moderate dementia = MMSE 10–19, severe dementia = MMSE < 10

Significance level: *p<0.05; **p<0.01

According to the new German S3 guidelines on dementia [16], there were *n* = 41 participants (19 urban, 22 rural) with MCI (i.e. MMSE > 26 & MoCA < 24), *n* = 107 participants (59 urban, 48 rural) with mild dementia (i.e. MMSE 20–26), *n* = 163 (113 urban, 50 rural) with moderate dementia (i.e. MMSE 10–19), and *n* = 30 (17 urban, 13 rural) with severe dementia (i.e. MMSE < 10).

### Comparison health services utilization for participants living in urban vs. rural SHAs

Table 3 shows the comparison health services utilization for participants living in urban and rural SHAs, divided into the five sections “Medication and vaccination”, “Inpatient/outpatient medical services”, “Non-pharmacological therapies according to German Remedies Directive”, “Health and medical aids”, “other important parameters”.

**Table 3:** Utilization of health services and health aids – urban vs. rural SHAs

Variable	Mean (SD) / *N* (Percentage) Urban (*n* = 208)	Mean (SD) / *N* (Percentage) Rural (*n* = 133)	*p*-value^a^	Corrected *p*-value^b^
***Medication and vaccination***				
Number of prescribed medications (sum)	6.91 (3.16)	8.40 (3.81)	< 0.001**	0.035*
Vaccinations received (yes)				
Influenza	76 (36.5)	48 (36.1)	0.806	0.986
Pneumococcus	15 (7.2)	10 (7.5)	0.988	0.991
***Inpatient/outpatient medical services***				
Hospitalization (in-patient) (last 6 months) (yes)	40 (19.2)	32 (24.1)	0.287	0.639
Out-patient hospital visit (last 6 months) (yes)	14 (6.7)	12 (9.0)	0.729	0.986
General practitioner visit (last 6 months) (yes)	194 (93.3)	128 (96.2)	0.243	0.578
Internal medicine specialist visit (last 6 months) (yes)	29 (13.9)	12 (9.0)	0.173	0.497
Gynecologist visit (last 6 months) (yes)	10 (4.8)	4 (3.0)	0.414	0.797
Surgeon visit (last 6 months) (yes)	13 (6.3)	8 (6.0)	0.930	0.991
Orthopedist visit (last 6 months) (yes)	9 (4.3)	6 (4.5)	0.935	0.991
Neurologist/Psychiatrist visit (last 6 months) (yes)	103 (49.5)	31 (23.3)	< 0.001**	0.035*
Dermatologist visit (last 6 months) (yes)	19 (9.1)	9 (6.8)	0.437	0.797
Ophthalmologist visit (last 6 months) (yes)	24 (11.5)	17 (12.8)	0.731	0.986
Urologist visit (last 6 months) (yes)	19 (9.1)	13 (9.8)	0.714	0.986
Dentist visit (last 6 months) (yes)	59 (28.4)	30 (22.6)	0.234	0.577
Psychotherapist visit (last 6 months) (yes)	8 (3.8)	4 (3.0)	0.682	0.986
Alternative practitioner visit (last 6 months) (yes)	4 (1.9)	3 (2.3)	0.833	0.986
***Non-pharmacological therapies according to German Remedies Directive*** ^***c***^				
Number of prescribed non-pharmacological therapies (sum)^c^	1.33 (1.18)	0.97 (0.90)	0.008**	0.079
***Health and medical aids***				
Use of walker (yes)	99 (47.6)	80 (60.2)	0.053	0.305
Use of wheelchair (yes)	55 (26.4)	45 (33.8)	0.144	0.497
Use of stair lift (yes)	17 (8.2)	12 (9.0)	0.784	0.986
Use of bathtub lift (yes)	12 (5.8)	11 (8.3)	0.369	0.759
Use of vision aids (yes)	139 (66.8)	92 (69.2)	0.651	0.986
Use of hearing aids (yes)	31 (14.9)	22 (16.5)	0.684	0.986
Use of dentures (yes)	121 (58.2)	80 (60.2)	0.717	0.986
Use of oxygen device (yes)	3 (1.4)	6 (4.5)	0.085	0.414
Use of sleep apnea treatment device (yes)	3 (1.4)	2 (1.5)	0.963	0.991
Use of compression stockings (yes)	60 (28.8)	42 (31.6)	0.591	0.986
Use of incontinence aids (yes)	132 (63.5)	86 (64.7)	0.822	0.986
Use of other medical aids (yes)	30 (14.4)	23 (17.3)	0.476	0.842
***Other important parameters***				
Infections requiring medical treatment (last 6 months) (yes)	58 (27.9)s	32 (24.1)	0.434	0.797
Number of falls (last 6 months) (sum)	0.63 (1.15)	0.66 (1.24)	0.843	0.986

^a^*p*-values for Chi-square tests for categorical variables and Mann-Whitney-U-Test for metric/interval-scaled variables

^b^*p*-values corrected for multiple testing by the Benjamini-Hochberg procedure

^c^Nonpharmacological therapies according to Remedies Directive (“Heilmittel-Richtlinie– HeilM-RL” version 18.04.2024: https://www.g-ba.de/downloads/62–492-3500/HeilM-RL_2024-04-18_iK-2024-07-23.pdf) including physiotherapy, medical chiropody, occupational therapy, speech/language and swallow therapy, other non-pharmacology therapies, e.g. nutrition therapy, applied by non-physician specialists

Significance level: *p<0.05; **p<0.01

Inhabitants of rural SHAs were prescribed a significantly larger number of medications (T (244) = −3.740; p_corrected_ = 0.035), while the provision of vaccinations were not significantly different across living areas.

Furthermore, urban inhabitants had seen a neurologist or a psychiatrist about twice as often in the last 6 months in comparison with rural inhabitants (χ² (1) = 23.365; p_corrected_ = 0.035), while the use of all other inpatient/outpatient medical services did not differ significantly.

Non-pharmacological therapeutic offers applied by non-medical specialists were prescribed in average not statistically significant more often in the last 6 months between the living regions (p_corrected_ = 0.079). The therapies most used were greatly similar: In urban regions, medical chiropody (54.8%) was used most frequently, followed by physiotherapy (30.8%), occupational therapy (25.5%), other non-medical therapies, e.g. nutrition therapy (13.5%), and speech/language/swallow therapy (8.7%). Medical chiropody (47.4%) was also most frequently used in rural regions, followed by physiotherapy (27.8%), occupational therapy (17.3%), other non-medical therapies, e.g. nutrition therapy (2.3%), and speech/language/swallow therapy (2.3%).

Beyond that, the prescription and use of health and medical aids as well as other important parameters such as falls and infections were not significantly different across living areas.

### Guideline adherence concerning anti-dementia medication in an urban-rural comparison

In summary, AChE inhibitors were prescribed to 18.5% (*n* = 63) of the participants, while Memantine was prescribed to 11.4% (*n* = 39) of the participants. Table 4 presents the urban-rural comparison of the frequency of guideline adherence/non-adherence for each of the nine test cases. There were no significant differences between rural and urban SHAs with regard to adherence/non-adherence to guidelines when prescribing anti-dementia medication.

**Table 4:** Urban-rural comparison of prescriptions for anti-dementia medication in accordance with the S3 guidelines on dementia

Variable (*n* of severity of cognitive impairment subgroup (urban/rural)	*N* (Percentage of subgroup) Urban	*N* (Percentage of subgroup) Rural	*p*-value^a^	Corrected *p*-value^b^
Acetylcholinesterase inhibitor for participants with mild dementia (*n* = 107 (59/48))	9 (15.3)	6 (12.5)	0.683	0.986
Acetylcholinesterase inhibitor for participants with moderate dementia (*n* = 163 (113/50))	27 (23.9)	13 (26.0)	0.773	0.986
Acetylcholinesterase inhibitor for participants with severe dementia (*n* = 30 (17/13))	5 (29.4)	1 (7.7)	0.141	0.497
Acetylcholinesterase inhibitor for participants with MCI (*n* = 41 (19/22))	2 (10.5)	0 (0.0)	0.119	0.479
Memantine for participants with moderate dementia (*n* = 163 (113/50))	14 (12.4)	6 (12.0)	0.944	0.991
Memantine for participants with severe dementia (*n* = 30 (17/13))	0 (0.0)	4 (30.8)	0.014*	0.107
Memantine for participants with mild dementia (*n* = 107 (59/48))	6 (10.2)	5 (10.4)	0.967	0.991
Acetylcholinesterase inhibitor with Memantine parallelly (*n* = 39 (22/17))	1 (4.5)	0 (0.0)	0.373	0.759
Memantine for participants with MCI (*n* = 41 (19/22))	2 (10.5)	2 (9.1)	0.877	0.991

^a^*p*-values for Chi-square tests

^b^*p*-values corrected for multiple testing by the Benjamini-Hochberg procedure

Significance level: *p<0.05

## Discussion

We wanted to use a hypothesis generating approach to explore whether there were inequities in diagnosing, medication and vaccination, utilization of inpatient/outpatient medical services, utilization of non-pharmacological therapies, the use of health and medical aids, guideline-adherent anti-dementia treatment, or SHA structures for PlwDs and PlwMCIs living in rural vs. urban SHAs across Germany.

Organized by our research questions, we found:

- *For the structural data of the SHAs*: no statistically significant differences in the qualifications of staff and the number of available staff, in the number of residents in each SHA, and in the implemented structural quality characteristics, such as the availability of dementia-specific training concepts as well as in the number of GPs serving the SHA.
- *For possible inequities in medication and vaccination*,* utilization of inpatient/outpatient medical services*,* utilization of non-pharmacological therapies according to the German Remedies Directive*,* and the provision of health and medical aids for PlwDs and PlwMCIs living in rural vs. urban SHAs across Germany*: a significantly larger total sum of prescribed medications for people living in rural locations and a significantly lower frequency of visits to a neurologist/psychiatrist for rural participants.
- *For the adherence to the recommendations of the “S3 guidelines on dementia”* [16] *concerning medical treatment of cognitive symptoms*: no significant differences in guideline-adherent and non-adherent prescription behavior concerning anti-dementia medication for PlwMCIs and PlwDs.

In total, only two statistically significant differences were found: people in rural SHAs were prescribed a larger number of medications and visited a psychiatrist/neurologist less often. There were no significant differences in GP visits, dementia diagnoses, the use of non-pharmacological therapies and health and medical aids, guideline-adherence concerning anti-dementia treatment, variables such as vaccinations, infections, falls, hospitalizations, or the use of a broad range of medical specialists. Also, there were no differences in staff availability, in the implementation of dementia-specific concepts, or in the size of the SHAs between urban and rural SHAs.

Thus, our results only partly reflected the results of the systematic review by Arsenault-Lapierre et al. [12], who reported that rural community-dwelling PlwDs had a lower number of visits to any physician (GP or specialist). In our study, this finding applied only to the category neurologist/psychiatrist. However, this difference could be a sign of an access barrier and constitutes an important difference concerning equity in location of residence because of the crucial role location plays in the diagnosis and treatment of PlwMCIs and PlwDs. Another possible explanation could be the finding, that in the present study, participants in rural regions were descriptively somewhat less cognitively impaired than those in urban regions. However, as the difference was statistically insignificant, this might not have been the main reason for the observed utilization difference.

Koller et al. [52] also found the same result we found in our study concerning urban-rural differences in consultations with neurologists/psychiatrists for community-dwelling PlwDs in Germany when analyzing claims data. In their study, PlwDs who resided in urban surroundings had a significantly higher chance (about 43% higher) of visiting a neurologist/psychiatrist (a “specialist”) in comparison with their rural-bound counterparts. Furthermore, in line with our results, Kosteniuk et al. [15] reported a lower probability of at least one specialist visit and a lower average number of specialist visits for PlwDs living in rural areas in Canadian administrative health data. Also, Xu et al. [13] identified a lower number of visits to a neurologist/psychiatrist in people with early-onset dementia living in rural areas in the US.

To explain the difference in (all types of) physician visits, the Canadian systematic review authors Arsenault-Lapierre et al. [12] proposed that there might be fewer professionals practicing in rural areas. One explanation for this phenomenon might be that rural areas are often perceived as less attractive due to inadequate infrastructure and limited economic opportunities. For example, Lu et al. [53] found that family medicine graduates in Canada tend to avoid practicing in rural areas due to concerns related to lifestyle and family, while Natanzon et al. [54] identified a lack of leisure facilities as a significant deterrent among German physicians. Furthermore, in Germany, GPs and specialists in ambulatory care are primarily self-employed and remunerated through capitation or fee-for-service schedules. This remuneration model may lead to a disadvantage for physicians in rural areas, as they may have fewer patients or provide a lower volume of services. Regional adjustments of payment or additional incentives beyond the standard systems could ensure sufficient financial motivation [55]. Another reason that fewer physicians practice in rural areas might be working conditions, as many medical students and recent graduates prioritize work-life balance when deciding where to work [56], and rural practice is often perceived as involving long hours and always being on call [57]. Actually, GPs or family physicians in Germany typically work more hours per week in rural settings compared with their urban counterparts [58]. Moreover, Creed et al. [59] found that Australian medical students perceive rural medicine as having low prestige and offering a less desirable lifestyle. In light of these findings, one might conclude that prestige may also be a factor that contributes to the smaller number of professionals practicing in rural areas in Germany.

Arsenault-Lapierre et al. [12] additionally hypothesized that, due to the lack of physicians, patients in rural areas of Canada had more visits to registered nurses by contrast. However, registered nurses are not available or authorized in all countries, as is the case in Germany. Thus, our data, which do not reflect significant regional differences in GP visits, correspond with the health system in Germany where the basic health care is GP-bound and thus GP-utilization is high. GPs in Germany often function as gatekeepers and care-coordinators who then suggest that patients be transferred to specialists. On the other hand, the availability and use of specialists in rural areas can be difficult in Germany, and thus, the interpretation that an access barrier exists might apply in this case. Large distances and thus more difficult accessibility have been reported as the main barriers [60], as a higher density of neuropsychiatric specialists are available in urban areas [61].

In contrast to Arsenault-Lapierre et al. [12], we did not find a larger number of hospitalizations for PlwDs or PlwMCIs living in rural areas, a finding that is in turn consistent with Kosteniuk et al.’s [15] recent study, which also did not identify any differences in hospitalization. These mixed results in the different studies suggest that, concerning hospitalization, additional factors beyond rural vs. urban locations may play a role. One factor might be that the participants living in rural areas in our study were not exclusively community-dwelling PlwDs but were already integrated into the medical and care assistance system. Additional factors might be the number and severity of comorbidities, dementia severity, and overall health status, as Gessert et al. [62] found higher hospital admission rates for people with severe dementia at the end of life living in rural nursing homes.

The fact that our study sample was already integrated into the medical and care assistance system may also be the reason why, in contrast to other studies [12, 13, 63, 64], we were unable to detect any significant urban-rural differences in terms of an existing dementia diagnosis. However, we found a descriptively larger number of participants with a dementia diagnosis in urban areas (79% vs. 67% in rural areas). Although other studies have differentiated between access to neuropsychological testing, psychological assessment, and imaging procedures, such data were not available to us. Indirectly, the lower number of visits to a psychiatrist/neurologist in our study could be a hint that there are potential differences in diagnostic procedures because either the specialists themselves carry out intensive diagnostic procedures or they coordinate the process through referrals.

In line with Arsenault-Lapierre et al. [12], we found that PlwMCIs and PlwDs in rural SHAs were prescribed a higher average number of medications compared with those in urban SHAs. One possible explanation for this finding could be that the rural inhabitants of our study had a slightly larger number of life-expectancy-relevant comorbidities at least on a descriptive level. Another interpretation could be the lower frequency of visits to a neuropsychiatric specialist by rural participants. Bohlken et al. [14] showed that the probability of receiving additional psychotropics (neuroleptics) as well as hypnotics/sedatives was significantly lower for PlwDs who were treated only by a neuropsychiatric specialist in comparison with those treated only by a GP.

The mixed results concerning anti-dementia medication reported by Arsenault-Lapierre et al. [12] were reflected in our results where no significant difference in the prescription of anti-dementia medication and guideline adherence was found. In total, 29.9% of participants in our study had an anti-dementia drug prescription, which was only slightly higher than the percentage reported in a large German claims study (24.6%) [14]. In this study mentioned by Bohlken et al., the majority of the anti-dementia prescriptions was, as in our study, mostly guideline-adherent, and differences between urban and rural areas were not relevant [14]. In some medication subgroups, however, our study revealed that medication was not administered in accordance with the guidelines in a few cases. Although the number of cases was small and the results should therefore be interpreted with caution, the observed deviations from the guidelines suggest that future surveys should also monitor guideline adherence in medication treatment in SHAs.

### Limitations

To our knowledge, our study is the first to explore urban-rural differences for PlwDs and PlwMCIs living in SHAs. However, when interpreting the data, some limitations need to be considered. First, even though we have recruited in all German federal states and included SHAs from the majority of all German federal states, this is not a sample drawn by chance. Therefore, our sample cannot be named representative of all German SHAs, because it was taken from a cluster-randomized trial, where SHAs were participating voluntarily. Thus, it is likely that our sample was very motivated, possibly “more open” towards research projects, and possibly better equipped, e.g. concerning the quantity of available resources in the SHA. Therefore, it is possible, that the data we report, might be an estimation that is more positive than the reality in German SHAs concerning use of different health care offers and that the utilization of health services could be overestimated compared with the population of all SHAs in Germany. However, such a trend would apply to both urban and rural SHAs. On the other hand, it is also possible that the differences between urban and rural areas in the population of all SHAs are much greater than shown in our data and are therefore underestimated due to possible recruitment bias.

Furthermore, the DemWG study was carried out during the COVID-19 pandemic. Thus, we cannot rule out that, during that time, barriers to the utilization of health care services outside the SHA were higher than in pre- or post-pandemic time frames. Such barriers could have led to a lower utilization of health services. However, the restrictions were uniformly applied to both rural and urban populations and were thus unlikely to have influenced the assessment of urban-rural differences.

In contrast to many other studies reporting on urban-rural differences, our study did not rely on claims data and therefore had a much smaller sample size. However, one advantage of our study is that we had primary data, which are more detailed and include additional health parameters of the patients as well as data on the locations of their residences.

Furthermore, the results reported here cannot be easily transferred to other countries with other healthcare systems and other forms of homelike care environments. Nevertheless, this is the first comprehensive analysis of urban-rural differences in health care for people with dementia and MCI in homelike care environments. Comparable innovative care environments also exist in other countries, e.g. “small-scale living arrangements” in the Netherlands, “green houses” in the USA, “group homes” in Japan, “Group Living” in Sweden, etc [6, 7]. Thus, the presented results could also be helpful and informative for other countries. In addition, they can provide relevant impulses for research on urban-rural differences in this particular setting that may also exist in other countries.

The study had a hypothesis generating character and explored a large number of variables. Thus, we needed to rigorously correct the alpha rate to prevent the consequences of inflating the alpha-error. A confirmatory analysis using claims data from individuals living in SHAs, focusing on variables such as drug prescriptions and neurologist/psychiatrist visits would be valuable.

## Conclusions

Most of the variables we explored did not show significant differences in the structure or the provision of staff in the SHAs, health services utilization, guideline-adherent anti-dementia medication, use of health and medical aids, vaccinations, falls, infections, or hospitalizations. Only two variables showed stable, significant differences, with PlwDs and PlwMCIs in rural SHAs being prescribed a larger total number of drugs and having a lower number of visits to a neurologist/psychiatrist. Although most variables indicated equity between rural and urban SHAs in our non-representative sample in the health care of PlwDs and PlwMCIs, the differences that were identified remain crucial. In particular, neurologists/psychiatrists play a significant role in providing concise diagnostic procedures– possibly mirroring in a descriptive urban-rural difference in the frequency of medical dementia diagnosis in our sample or in supporting referrals to different type of specialists. Even under consideration that we might have recruited a selected sample, the inequal use of neurologists/psychiatrists reflected in our data could be the one crucial remaining inequity between living regions and lead to further differences in medical diagnostics and care. It is a matter of fact, that access to such essential specialists is more challenging in rural areas. Recent studies have suggested that “mobile diagnostic centers” could be provided to overcome these challenges in rural areas [60]. Possibly that suggestion would not only improve diagnostic procedures but also the referral practices or even the provision of different care offers.

## Supplementary Information

Supplementary Material 1.

## Data Availability

The data sets generated or analyzed during the current study will be available upon request from Stephan Kloep (kloep@uni-bremen.de). Data will be available in the time interval from 12 to 36 months after the article is published. The data will be provided for non-commercial research purposes only to researchers with a proposal that was peer-reviewed and approved by an independent review committee. Interested researchers must present an analysis plan and state the research purpose for which the data are needed (e.g. meta-analysis). Data will be available through the data warehouse of the University of Bremen without any additional investigator support. The data that can be provided refer solely to the data underlying the results presented in the manuscript. Data will be completely anonymized, and access to the stored data with personal information will not be possible. Thus, case-specific additional information/clarification can no longer be provided.

## Abbreviations

ADLs: Activities of Daily Living
BPSDs: Behavioral and Psychological Symptoms of Dementia
CCI: Charlson Comorbidity Index
CMAI: Cohen-Mansfield Agitation Inventory
CMAI-SF: Cohen-Mansfield Agitation Inventory-Short Form
CRF: Case Report Form
FIMA: Questionnaire for health-related resource use in an elderly population
GPs: General Practitioners
KKSB: Competence Center of Clinical Trials of the University of Bremen
M: Arithmetic Mean
MCI: Mild Cognitive Impairment
Mdn: Median
MMSE: Mini-Mental State Examination
MoCA: Montreal Cognitive Assessment
NPI: Neuropsychiatric Inventory
NPI-NH: Neuropsychiatric Inventory-Nursing Home Version
PlwDs: People living with Dementia
PlwMCIs: People living with Mild Cognitive Impairment
QoL: Quality of Life
SD: Standard Deviation
SHAs: Shared-Housing Arrangements
