# Supplementary material for: Urban-rural differences in the health care of people living with dementia and mild cognitive impairment in shared-housing arrangements in Germany – have inequities in urban vs. rural locations been overcome?
Source: BMC Health Serv Res. 2025 Mar 13;25:371. doi: 10.1186/s12913-025-12508-z (PMC11905690; doi:10.1186/s12913-025-12508-z)
Supplement: Supplementary file 1 — Supplementary Material 1. [file 12913_2025_12508_MOESM1_ESM.docx]

How many people with dementia or mild cognitive disorder live in your SHA?

Are you an SHA specialized on intensive care or on mental disorders other than chronic progredient cognitive diseases (like dementia)?
